# Editorial: Applied bioinformatics in insect physiology

**DOI:** 10.3389/fphys.2024.1537476

**Published:** 2024-12-18

**Authors:** Herbert Venthur, Joel Vizueta, Jesus Lozano-Fernandez

**Affiliations:** ^1^ Laboratorio de Química Ecológica, Departamento de Ciencias Químicas y Recursos Naturales, Facultad de Ingeniería y Ciencias, Universidad de La Frontera, Temuco, Chile; ^2^ Centro de Investigación Biotecnológica Aplicada al Medio Ambiente (CIBAMA), Universidad de La Frontera, Temuco, Chile; ^3^ Department of Biology, Villum Centre for Biodiversity Genomics, Section for Ecology and Evolution, University of Copenhagen, Copenhagen, Denmark; ^4^ Departament de Genètica, Microbiologia i Estadística and Institut de Recerca de la Biodiversitat (IRBio), Facultat de Biologia, Universitat de Barcelona (UB), Barcelona, Spain

**Keywords:** insect physiology, bioinformatics, gene expression, pests, invertebrates, high throughput sequencing, gene function

## Introduction

Since the early 2000s, understanding gene function in invertebrates has become approachable through genomics (Smith and Eyre-Walker, 2002; Clark et al., 2007). Some years later, high-throughput DNA and RNA sequencing became available in the frame of the development of next generation sequencing technologies, allowing comparative genomics studies (Berens et al., 2015). In parallel, the prediction of protein structure through molecular modelling techniques, was progressing thanks to the obtention of crystallized 3D structures (e.g., Kuhlman and Bradley, 2019). Overall, genomic research started to emerge around invertebrate and especially insect physiology, which has been heavily supported by bioinformatics analyses in recent years (see for example, Vizueta et al., 2020; Venthur and Zhou, 2018; Chen et al., 2022).

Nowadays, applied bioinformatics at sequence and structural level have helped the exploration of key physiological processes in insects, particularly those that have become pests due to globalization, intensive agriculture and/or climate change. Thus, genomics and transcriptomics have provided extensive datasets to explore gene function, such as chemosensory proteins and enzymes for biosynthesis or detoxification in insects, crucial for their life cycles. Therefore, the objective of this Research Topic was to present and discuss research around insects and how applied bioinformatics represents an essential tool to explore gene function, using phylogenetic analysis, protein structure prediction, or molecular evolution methods, among others. Likewise, newly developed methods and protocols that fit the needs of insect physiology were welcome in order to provide novel valuable resources for the research field.

Overall, this Research Topic showcased studies into the molecular mechanisms governing the physiology of insects by means of bioinformatic tools. It integrates articles using perspectives from physiology, gene expression analyses and bioinformatics, offering a comprehensive understanding of the physiology of insects.

## Research topic

Dong et al. have characterised the transcriptome profile of the final leg segment, the tarsi, of the fall armyworm moth *Spodoptera frugiperda.* This species is a serious moth pest that can damage many plants worldwide. The tarsus is the first body part in contact with the plants which these organisms feed on, and provide these moths with the ability to detect chemical signals which are important for food detection. The authors analysed the gene expression of this body part, annotated putative chemosensory receptor genes, and used quantitative PCR to compare the expression of these genes in different chemosensory organs, as the antennae. They found that few chemosensory receptors were enriched in the tarsi regions, suggesting an important role in tarsal contact chemosensation of food.

Sheraz et al. presented a study on a major rice pest, the brown planthopper *Nilaparvata lugens*. There is an ongoing global effort to understand the causes of resistance in seasonal pests to pesticide usage. The authors focused on the superoxide dismutase gene family, which is involved in the response to pesticides. They classified the sequences from this species into three subgroups, and evaluated their gene expression by quantitative PCR. The authors found that after exposure to stress, the gene expression levels of these gene families vary, suggesting their potential role in modulating the development of these species when exposed to pesticides.

Hou et al., instead, focused on an emerging pest on legume crops, the thysanoptera *Megalurothrips usitatus*. Molecular studies on this species have been scarce, and the authors made a first attempt to find stable reference genes (housekeeping) for conducting quantitative PCR studies. They concluded that *Actin* and the *Ribosomal protein L* were the most suitable, as they remained stable after different stressors. Furthermore, the authors tested those housekeeping genes to evaluate the expression levels of cytochrome P450 gene (CYP450) in different experimental conditions. Overall, their study provides a basis to evaluate gene expression by quantitative PCR in this species.

Finally, Tan et al. addressed the effect of essential oils, an alternative to insecticides, to control the populations of the tea green leafhopper, *Empoasca onukii,* which is one of the most devastating pests in the Chinese tea industry. Studies regarding the effect of plant essential oils on this species are scarce, and the authors performed comparative transcriptome analyses to fill this gap. They found that essential oils caused toxicity in this species, and that many of the differential expressed genes after the treatment contain detoxification genes and redox-related gene families, suggesting that these might be involved in the response to oil stress. This study offers new insights for the management of this pest in the future.

## Conclusion

This series of papers offers new developments in the field of insect physiology, particularly for applied research, and how bioinformatics and the comprehensive study of gene expression is the *status quo*. They demonstrated that the field is active and in constant expansion to adapt to newer methodologies. Overall, they show that applied bioinformatics have a key role in understanding gene functions and the processes governing insect physiology.
